# Supplementary material for: Ensemble-based deep learning improves detection of invasive breast cancer in routine histopathology images
Source: Heliyon. 2024 Jun 14;10(12):e32892. doi: 10.1016/j.heliyon.2024.e32892 (PMC11252882; doi:10.1016/j.heliyon.2024.e32892)
Supplement: Multimedia component 1 [file mmc1.docx]

**Ensemble-based deep learning improves detection of invasive breast cancer in routine histopathology images**

Leslie Solorzano, Stephanie Robertson^2^, Balazs Acs^2^, Johan Hartman^2^, Mattias Rantalainen^1^

^1^Department of Medical Epidemiology and Biostatistics, Karolinska Institutet, Stockholm, Sweden

^2^Department of Oncology-Pathology, Karolinska Institutet, Stockholm, Sweden

#

# Supplementary material

| 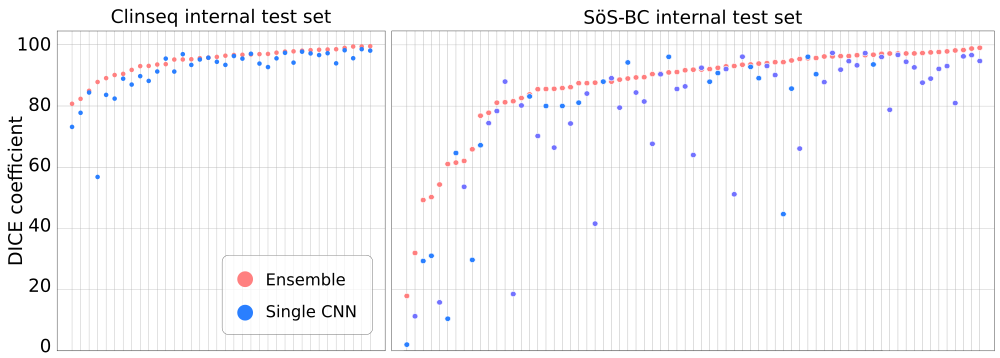 |
| --- |
| Figure S1. **Dice coefficients in internal test sets.** Pink markers show the dice coefficient achieved by the ensemble in the internal test sets for each WSI. Blue markers show the results of the single network. At a glance the large number of blue markers under the pink area shows that the ensemble achieved a higher proportion of accuracies per WSI. |

| 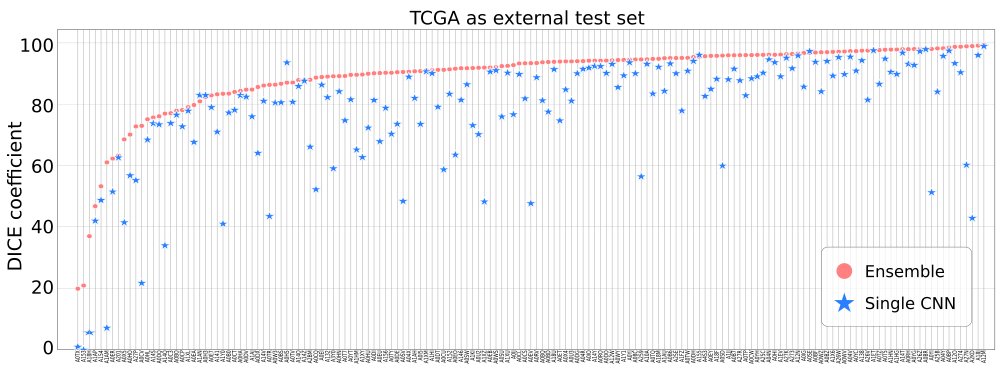 |
| --- |
| Figure S2. **Dice coefficients in the external test set.** Pink markers show the dice coefficient achieved by the ensemble in the TCGA dataset, for each WSI. Blue markers show the results of the single network. At a glance the large number of blue markers under the pink area shows that the ensemble achieved a higher proportion of dice coefficients per WSI. |

| 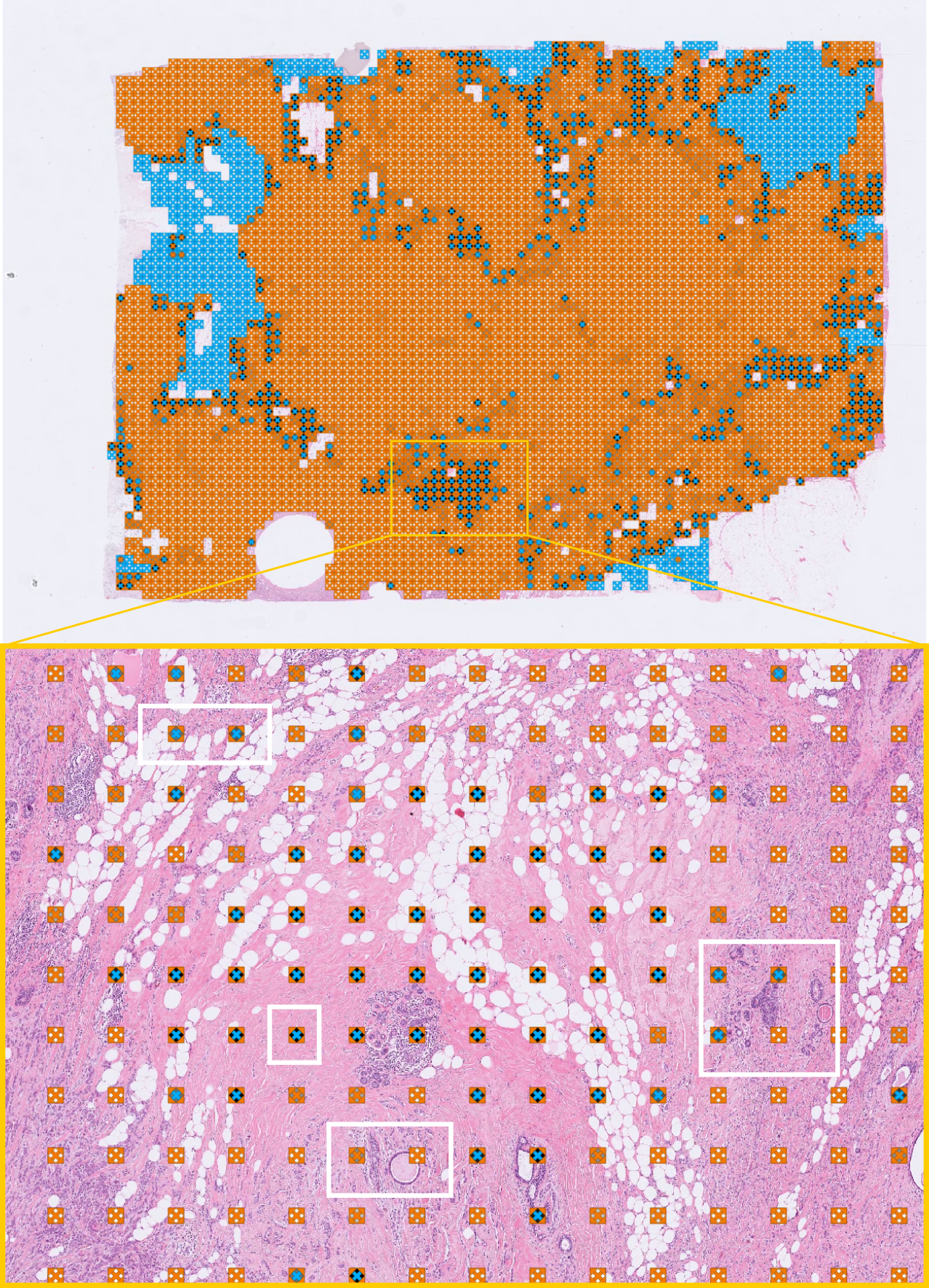 |
| --- |
| Figure S3. Agreement on WSI where a single model surpassed the ensemble accuracy. We want to explore why this could be. Note the agreement of lower than 10 (gray colors) along the border of a big IC region (orange). An enlarged area is highlighted and the tissue underneath is observed, note that there is adipose tissue present and structures marked in white. |

| 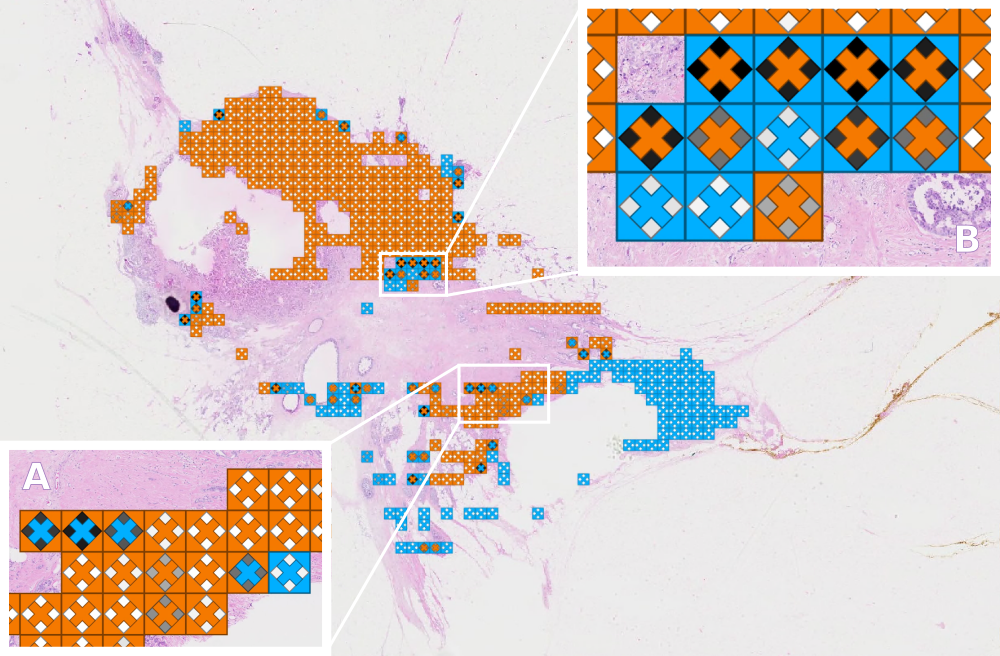 |
| --- |
| Figure S4. Visualization of agreement of a WSI where the difference in accuracy is small: 87.5% vs 83.56%. In A) a few tiles contain IC as ground truth while the predicted value is non-IC, with a gray agreement meaning it disagreed but not extremely. There are also some correctly labeled IC where not all CNN voted for IC. In B) a strong disagreement between labels indicates that the CNNs vote for IC, however the ground truth indicates non-IC. This kind of tile can be reviewed by humans. |
